# COVID-19: relationship between atmospheric temperature and daily new cases growth rate

**DOI:** 10.1017/S0950268820001831

**Published:** 2020-08-19

**Authors:** A. Rouen, J. Adda, O. Roy, E. Rogers, P. Lévy

**Affiliations:** 1 Département de Génétique Médicale, unité INSERM U933, Hôpital Armand-Trousseau, Assistance Publique-Hôpitaux de Paris, Paris, France; 2 Département de Cardiologie, CHU Montpellier, Montpellier, France; 3 Synlab Paris, Synlab France, Paris, France; 4 Departement de Santé Publique, Institut Pierre-Louis de Santé Publique (INSERM UMR S 1136, EPAR Team), Sorbonne Université, Assistance Publique-Hôpitaux de Paris, Hôpital Tenon, 75020 Paris, France

**Keywords:** Atmospheric temperature, COVID-19, seasonality

## Abstract

Purpose: The novel coronavirus (severe acute respiratory syndrome coronavirus-2 (SARS-CoV-2)) first appeared in Wuhan, China, in December 2019, and rapidly spread across the globe. Since most respiratory viruses are known to show a seasonal pattern of infection, it has been hypothesised that SARS-CoV-2 may be seasonally dependent as well. The present study looks at a possible effect of atmospheric temperature, which is one of the suspected factors influencing seasonality, on the evolution of the pandemic. Basic procedures: Since confirming a seasonal pattern would take several more months of observation, we conducted an innovative day-to-day micro-correlation analysis of nine outbreak locations, across four continents and both hemispheres, in order to examine a possible relationship between atmospheric temperature (used as a proxy for seasonality) and outbreak progression. Main findings: There was a negative correlation between atmospheric temperature variations and daily new cases growth rates, in all nine outbreaks, with a median lag of 10 days. Principal conclusions: The results presented here suggest that high temperatures might dampen SARS-CoV-2 propagation, while lower temperatures might increase its transmission. Our hypothesis is that this could support a potential effect of atmospheric temperature on coronavirus disease progression, and potentially a seasonal pattern for this virus, with a peak in the cold season and rarer occurrences in the summer. This could guide government policy in both the Northern and Southern hemispheres for the months to come.

## Introduction

The novel coronavirus (severe acute respiratory syndrome coronavirus-2 (SARS-CoV-2)) emerged in Wuhan, China, in December 2019 and rapidly spread globally, being officially termed a pandemic by the World Health Organization (WHO) on 11 March 2020 [1, 2]. As of 15 June 2020, 8 035 234 people worldwide have tested positive for the virus and 436 380 deaths caused by the virus have been recorded [3]. Despite the large number of publications on the subject (24 468 referenced publications on PubMed with the term ‘COVID’ as of 15 June 2020), many of the factors influencing the transmission and contagiousness of the virus remain elusive.

Infections caused by many respiratory viruses are known to be seasonal [4]. They are responsible for 2.6 million deaths each year, occurring mostly during the winter season. For this reason this period of the year is referred to as ‘cold and flu’ season in temperate climates. Several hypotheses have been proposed to explain this relationship, concerning both the pathogens and the hosts. It has been hypothesised that coronavirus disease 2019 (COVID-19) could follow a similar seasonal pattern [5].

Seasonal patterns in infectious diseases are still to this day a little understood phenomenon, with many possible intertwined factors. Some are related to the pathogen itself, some are related to the host and some are related to the environment. Furthermore, and this is especially true for the first months of an epidemic, patterns can be observed without any causality of weather or seasonality. These confounding factors add to the complexity of identifying a seasonal pattern for a given pathogen. In this study, we used atmospheric temperature as a simple, easily available proxy, for seasonality – while keeping in mind that rules affecting viral transmission are overwhelmingly complex and sometimes even poorly understood.

The present study examines the correlation between atmospheric temperatures and the emergence of new COVID-19 cases, using data from nine different outbreak locations across four continents, associated with a broad array of conditions (differing in weather, governmental measures such as lockdown implementation or contact tracing etc.)

## Materials and methods

We conducted an observational retrospective study. We first collected publicly available data from weather reports (meteorology database Custom Weather, CA, USA) from nine different outbreak locations in four continents, from 1 January to 17 April 2020: Lombardy (Italy), London (United Kingdom), Ile-de-France region (France), Grand Est region (France), Stockholm (Sweden), Tehran (Iran), New York City (USA), Seoul Capital region (South Korea) and New South Wales state (Australia). Maximal temperatures in degrees Celsius (°C) were collected for each day. To limit bias and temperature uncertainties, relatively small regions were studied.

Secondly, we assembled publicly available data from official agencies (Italy: *Protezione Civile* daily bulletins, France: *Agence Santé Publique France*/French Health Ministry, United Kingdom: NHS England, Sweden: *Folkhälsomyndigheten* bulletins, Iran: Ministry of Health and Medical Education, USA/New York: New York State Department of Health, South Korea: Korea Center for Disease Control, Australia: Australian Government Department of Health) for new COVID-19-related diagnoses, over the same period of time, in the same locations. Instead of using absolute values, we used percentage of new diagnoses compared to the cumulative total of the day before, which we defined as the ‘growth rate’ of daily new cases. One however has to keep in mind that testing policies, and therefore case detection, can vary drastically between location, and even over time for a given location. However, since each outbreak was studied independently, we considered this did not constitute a systematic bias.

**Fig. 1.**
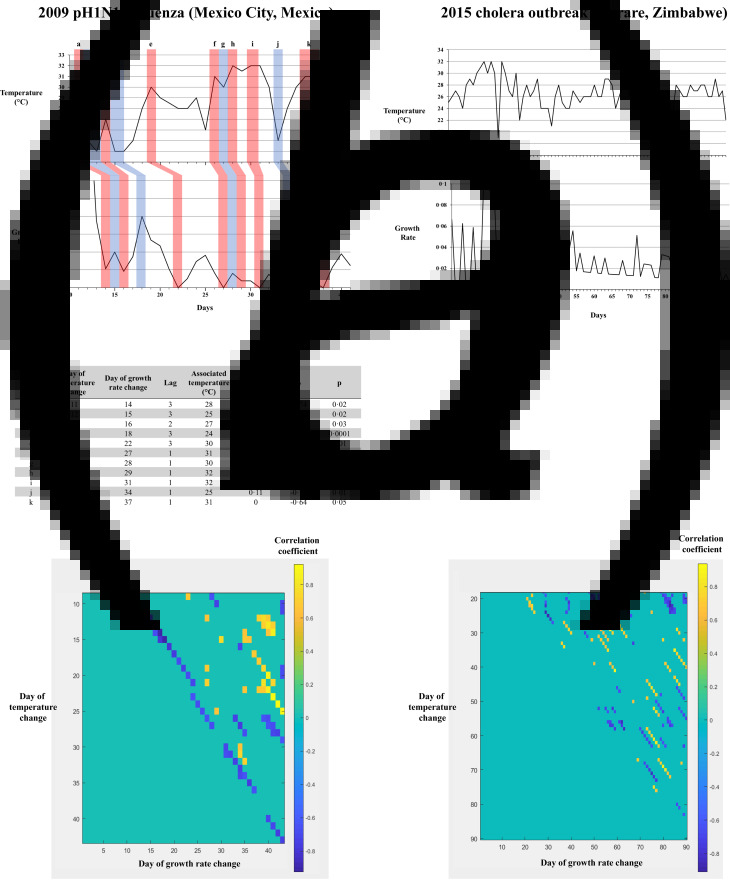
Day-to-day micro-correlation analysis validation, on the 2009 pH1N1 influenza outbreak in Mexico City, Mexico (positive control) and on the 2015 cholera outbreak in Harare, Zimbabwe. (a) Analysis of the pH1N1 outbreak in Mexico City shows negative-correlation sequences between temperature and growth rate, with a lag of 1−3 days. The generated matrix shows a long streak of negative correlation (in blue), with a lag considered to be constant. (b) Analysis of the cholera outbreak in Harare shows no evident correlation sequences. The generated matrix shows short streaks of negative (blue) and positive (orange) correlations, of various lag values (1−40 days), and seemingly randomly positioned.

For each change in temperature, positive or negative, the daily new cases growth rate curve was examined. Micro-correlation analysis was performed using a 10-day shifting window computing the Spearman correlation coefficient applying a one-day lag. This generated a matrix of all overlapping correlations with their corresponding *P*-values. Thus, a matrix was generated, showing *ρ* and *P*-values for day-to-day micro-correlations between atmospheric temperature and daily new cases growth rate, for each of the nine outbreaks (Fig. 1). As a positive control for this method, we analysed the first weeks of the 2009 pH1N1 influenza pandemic, despite its appearance in the spring later appeared to have a seasonal pattern [6, 7]. As a negative control we analysed 100 days of the 2015 cholera outbreak in Zimbabwe, which is known to follow a rather different pattern, with a very different relationship to weather [8].

Each outbreak location was studied independently. Correlation between the number of temperature curve deflections and daily new cases growth rate curve deflections was assessed with a Spearman rho test. Statistical analysis was performed using Matlab R2018a and Statview 5.0 (SAS institute). The results with *P* < 0.05 were considered to be statistically significant.

## Results

To validate our statistical method, we first analysed results for the two controls (Fig. 1).

As a positive control for this method, we analysed the first weeks of the pH1N1 influenza pandemic, which despite its initial appearance in the spring, later appeared to exhibit a seasonal pattern. We were able to identify long streaks of negative correlation with a constant lag between temperature and growth rate, which translated to temperature/growth rate sequences. The mean lag was 1.8 days (median: 1 day, standard deviation: 0.98 days). We concluded that there was an effect of atmospheric temperature on pH1N1 influenza growth rate, in the few weeks of the pandemic in Mexico.

As a negative control, we analysed 100 days of the 2015 cholera outbreak in Zimbabwe, and did not find any relationship sequences between temperature and growth rate. The generated matrix did show short streaks of negative correlations, but the lag was not compatible was pathophysiological basis, nor was it constant (mean lag: 20.4 days, median lag: 18 days, standard deviation: 15.1 days). Furthermore, there was an equal number of streaks of positive correlations, suggesting an artefactual and random phenomenon. We concluded that atmospheric temperature did not directly influence cholera growth rate on a day-to-day basis, in the 2015 outbreak in Zimbabwe.

**Fig. 2.**
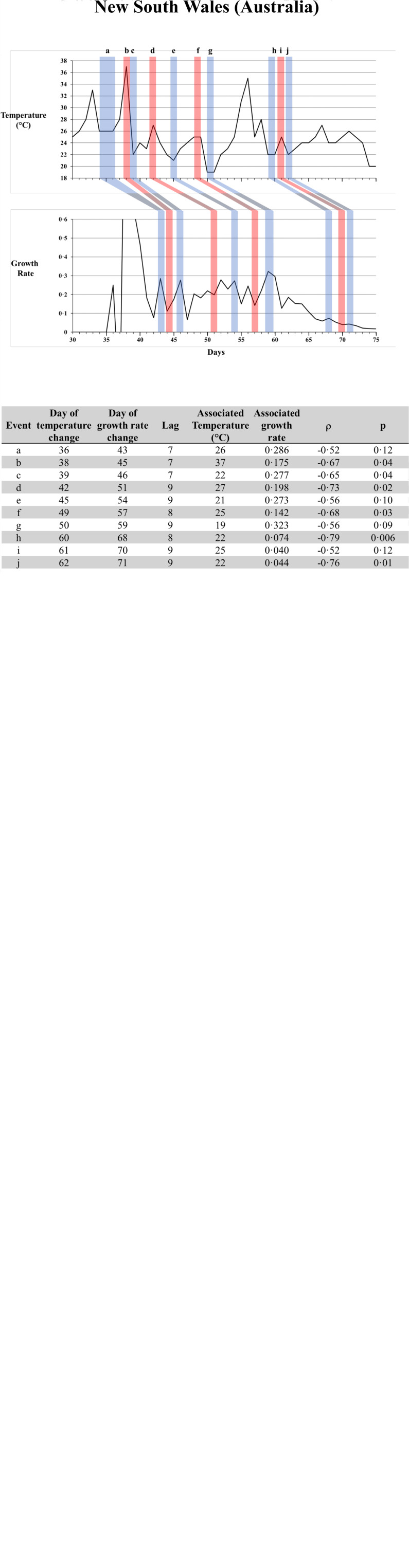
Atmospheric temperature and daily new COVID-19 cases growth rate variations, starting from the first day with COVID-19 in the location, in nine locations. Red bars highlight spikes in temperature and their associated relationship with growth rate, and blue bars highlight drops in temperature and their associated relationship with growth rate. The tables list the events (temperature spikes and drops, with the associated opposite variations in growth rate), with the day and temperature (°C) of the temperature change, the day and growth rate of the growth rate variation, the correlation coefficient (*ρ*) and *P*-value.

We then analysed data for all nine COVID-19 locations (Fig. 2). We show that several of the observed atmospheric temperature spikes were followed by a decrease in the daily new cases growth rate. Reciprocally, several of the observed drops in atmospheric temperature were followed by an increase in daily new cases. This led to a negative day-to-day micro-correlation between atmospheric temperature and the daily new cases growth rate, with a mean lag of 9.7 days.

We were able to identify a total of 49 events (spikes or drops in temperatures that were followed by a decrease or an increase in the daily new cases growth rate) that were statistically significant, in addition to 24 that were close to statistical significance, but were included nonetheless since they were part of sequences of relationships between temperature and growth rate. Those events were identified on the graphs and validated on the generated matrix, with associated *ρ* and *P*-values.

The mean lag between the temperature change and the variation in daily growth rate was 9.7 days (median: 10 days). The outbreak with the longest lag was in Seoul, South Korea (12.1 days) and the one with the shortest was in London (7.2 days).

While the lag differed between the outbreak locations, it was consistent for a given location. The mean global standard deviation was 0.79 days, with values ranging from 0 (New York City) to 1.55 (Seoul).

**Fig. 3.**
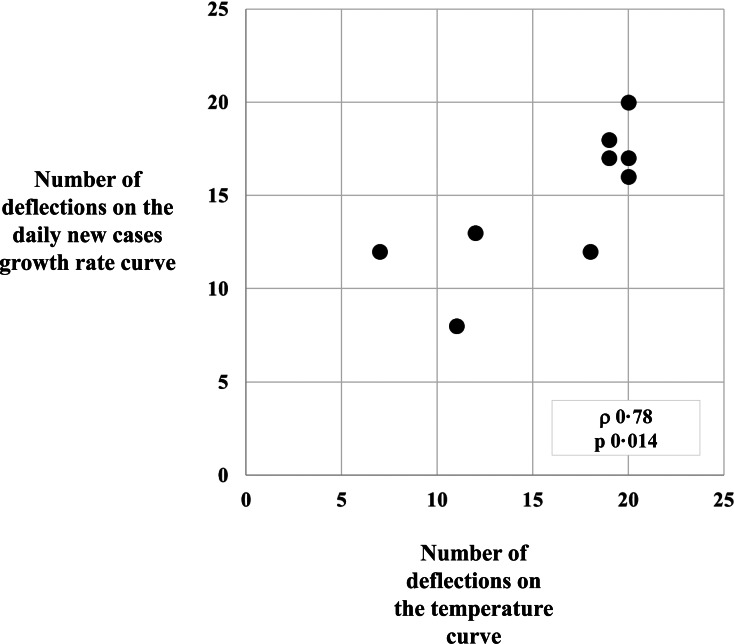
Global analysis, on all nine locations, of the number of deflections on the atmospheric temperature curves and of number of deflections on the daily new cases growth rate curves shows a positive correlation (*ρ* = 0.78, *P* = 0.014).

We then looked at the respective number of deflections in the temperature and growth rate curves, in all nine locations. For each location, the first recorded days show drastic, thus potentially misleading, variations in the new cases growth rates, owing to the small number of cases. In order to avoid incoherent results, we did not include those first few days in our analysis, which we began starting from 6 days after the first day that was associated with a growth rate <0.6, in order to eliminate the initial artefactual drastic variations. We found a positive correlation between the number of deflections in the temperature curves to the growth rate curve, with a lag of 10 days and for a length of 30 days (*ρ* = 0.78, *P* = 0.014, Fig. 3). The more changes (deflections) there were in the temperature curve, the more deflections could be observed in the daily new cases growth rate curve.

## Conclusion

Since the novel coronavirus SARS-CoV-2 emerged in Hubei Province, China, and spread to most countries across the globe, the factors influencing its transmission have been under intense scrutiny and produced a great deal of speculation.

It has been suggested that increased temperatures in the spring and summer would slow down the transmission of the virus and lessen its impact. Indeed, most respiratory viruses, including other coronaviruses, showcase a seasonal pattern of infection with outbreaks during colder months [4]. However, this had previously not been demonstrated for SARS-CoV-2.

In the present study, we collected atmospheric temperature data from weather reports from nine COVID-19 outbreaks across four continents in both hemispheres. Traditional statistical tools used to evidence seasonal patterns in epidemic phenomena, such as distributed lag non-linear models, were not the evident option, because of how recent the pandemic is. Such analyses look at relative risks over a year to show seasonal patterns. Considering the high number of confounding factors, most importantly, the lockdown measures implemented in many countries, we did not attempt to show a global correlation (linear regression) between the curves. Instead, we opted to conduct a day-to-day micro-correlation analysis. We looked for streaks of negative correlation values and significant *P*-values on a matrix that showed day-to-day correlations between atmospheric temperatures and new daily cases growth rates. While confounding factors undoubtedly blurred this relationship, we were able to evidence a negative correlation between temperature and new daily cases growth rate for all nine outbreaks with an average lag of 9.7 days.

In order to validate this novel seasonality evaluation method, we included in our analysis as a positive control the first weeks of the pH1N1 influenza pandemic in Mexico. While influenza epidemics are seasonal by nature, with outbreaks in the cold season, the pH1N1 pandemic broke out in the spring. Several months had to pass to evidence that it followed a similar pattern to that of other influenza strains, with recurrent outbreaks in the winter [7]. With our described method, we were able to evidence an influence of temperature on virus growth rate, which might reflect seasonality, after only a month and a half of observations.

As a negative control, we chose to analyse the 2015 cholera outbreak in Zimbabwe. The effects of the environment on cholera outbreaks are complex. Although cholera is not *per se* a seasonal disease, it may be influenced by seasonal factors. It is now known that *Vibrio cholerae* is riverine and thrives in coastal waters [9], and that its outbreak patterns are related to the ecology of the bacterium in the environment, with increases in cases in times of high water temperature and zooplankton blooming [10]. At the scale level of several years (1998–2016), Asadgol *et al*. found a positive correlation between cholera cases, high temperature and low precipitation [11]. However, the proposed pathophysiological model is that drought leads to bacterial multiplication, contamination of groundwater and increased use of untreated water (such as from wells) by the population. This is consequently a ‘large-scale’ correlation, with systemic causes and consequences occurring over several months, which explains the absence of evident day-to-day streaks of positive correlations in our day-to-day micro-analysis. We however did not find, as stated in the results, any evident sequences of negative correlations, as opposed to what was found in pH1N1 and ultimately COVID-19, which is coherent with our analysis.

Recently, Yao *et al*. studied the effect of temperature, humidity and UV radiation on COVID-19 propagation in 224 Chinese cities, and did not find any correlation [12]. However, Yao and colleagues specifically examined global correlations. When we performed the same analyses, we found similar results (figure shown in appendix). Considering the short length of the period of interest (several weeks, as opposed to a full year), and the overwhelming impact of confounding factors, we suggest that global correlation analyses are not the appropriate statistical analyses here. Instead, we developed a method to look at micro-correlations between atmospheric temperatures and virus propagation on a day-to-day basis. Some of those negative correlations did not fall within our standards for statistical significance (24 out of the 73 events are shown in Fig. 2). Nevertheless, we still chose to include these, since they were visually evident and were part of sequences containing other statistically significant negative correlations.

Many other parameters in addition to atmospheric temperature are at play and are certain to influence SARS-CoV-2 transmission. Of critical relevance is the effect of governmentally determined responses which have varied greatly among countries in both scope and effect. With initial exponential growths in the number of new cases and COVID-19-related deaths, governments in many countries have acted to implement unique sets of lockdown measures. The Chinese authorities imposed a lockdown on the Hubei Province on 23 January 2020, which is believed to have significantly reduced the transmission of the virus [13]. Other countries such as Italy, Spain and France, have since adopted similar measures.

It is of interest to examine the results in countries that did not implement strict lockdown measures, such as South Korea [14]. There, the authorities opted for a strategy based on large-scale testing (1 018 214 tests as of 8 June 2020), isolation of patients testing positive and intensive contact tracing. Those measures, taken together, led to a rapid decrease of the viral growth rate, and a relatively modest number of infected patients (12 121 in the whole country, as of 14 June 2020, according to the Korea Centre for Disease Control). Even under those conditions, we still evidence a correlation between atmospheric temperature and SARS-CoV-2 transmission. Conversely, countries such as France and Italy imposed strict lockdowns on their populations, which presumably decreased the virus's reproduction rate. In those countries as well, despite the prominent effect of the lockdown measures on the daily new cases growth rate, the effect of temperature changes remains evident.

Another outbreak of particular interest is that of Sweden. Unlike other countries facing the pandemic, the Swedish authorities chose not to implement any lockdown or phone-based contact tracing, relying instead on voluntary isolation measures. Again, in those conditions, we found a relationship between temperature variations and viral propagation.

In the analysis of the influence of temperature on viral propagation, the subject of countries within the temperate zone of the Southern Hemisphere is of interest. The summer and autumn conditions in those areas are thought to have potentially limited viral transmission so far. Indeed, after 4.5 months since the first cases in Australia, the number of cases and deaths (7335 and 102 respectively, as of 15 June 2020) is low. Because the authorities have imposed preventative measures, such as a strict lockdown of New South Wales beginning 31 March, it is difficult to determine the influence of atmospheric temperatures on the relatively mild aspect of the outbreak in the country. However, even in those conditions, we were able to showcase the relationship between atmospheric temperature and new cases.

The lag value varied between the nine outbreak locations, from 7.2 days in London to 12.1 days in Seoul, with a mean of 9.68 days. Descriptions conducted so far have suggested a mean incubation period of 5.2 days, with the 95th percentile of the distribution at 12.5 days [15]. Our findings are in accordance with those elements. The temporal variation seen here may be explained in part by variations in incubation time that are already known to exist [16]. Variations between lag times of the different locations could be explained by numerous factors, such testing policies and availability in individual countries, or reporting time and procedure.

Several hypotheses have been proposed to explain the relationship between atmospheric temperature, seasonality and viral transmission, with respect to both the pathogen and the host. For example, it has been suggested that warmer temperatures could decrease the stability of enveloped viruses such as coronaviruses by affecting the ordering of phospholipids [17]. Additionally, it has been shown that colder and drier air could affect airway passages, compromising ciliary function and mucus production [18]. Temperature influence on locally inducible innate antiviral immunity could also play a role. Indeed, it has been shown that rhinoviruses replicate faster in host cells at low temperature, owing to decreased type 1 IFN production from epithelial cells [19]. Finally, there are behavioural effects to consider. The cold season is associated with a ‘crowding effect’, where people gather indoors in poorly ventilated spaces. A precise relationship between a drop in temperatures in the winter and a peak in influenza infection rates has been shown by Azziz-Baumgartner *et al*. [20] Notably, the influenza virus in this study does not exhibit a seasonal pattern in the tropics, where there are much narrower temperature variations. As already mentioned, while the pH1N1 influenza A pandemic broke out in the spring and summer of 2009, it now follows a seasonal pattern, with epidemics in the winter [7]. This phenomenon has also been confirmed on animal models, with lower temperatures increasing the rate of influenza transmission among guinea pigs [21].

A relationship between humidity and viral transmission has also been suggested. Indeed, at high humidity, virus-filled droplets are rapidly eliminated from the air by a process of gravitational settling. Conversely, dry air leads to the formation of light dry aerosols that are able to float in the air for longer periods [22]. However, there is a complex relationship between temperature and humidity, and we chose only to study temperature in this paper because of the overwhelming confounding factors involved.

Similar seasonal patterns have been evidenced for other respiratory viruses, such as the respiratory syncytial virus (RSV), human metapneumovirus (hMPV), human parainfluenza virus type 1 and 2 (HPIV-1, HPIV2) and rhinovirus [23–25].

Similarly, coronaviruses in general seem to follow a seasonal pattern. Referencing the 2003 SARS outbreak, Tan *et al*. in 2005 reported a significant correlation between SARS-CoV incidence and weather conditions 7 days before the outbreak, showing the potential effect of a sharp change of temperature on virus transmission [26]. Additionally, Lin *et al*. in 2006 observed a significant decrease in the SARS-CoV incidence as temperatures increased from 15 °C to 29 °C [27]. Gardner *et al*. showed similar findings during the outbreak of Middle East respiratory syndrome coronavirus (MERS-CoV), which occurred from 2015 to 2017 [28]. Other human coronavirus (HCoV), such as strains HKU1, OC43, 229E and NL63 also follow a seasonal pattern, with outbreaks in the cold season [29].

The assumption that an influence of atmospheric temperature equals seasonality is complex. It is therefore worth mentioning that seasonality cannot be limited to the simple effect of the weather. Many other factors, some of them yet to be elucidated, are at work in causing seasonal patterns in certain epidemic outbreaks. We here show that variations in atmospheric temperature might affect COVID propagation, on a very fine scale basis. This could suggest a future seasonal pattern, which is still to be confirmed.

Do these results mean that the COVID-19 pandemic will decline in the summer in the Northern Hemisphere, in temperate-climate latitudes? We show that changes in temperature are followed by opposite changes in the daily new cases growth rate. However, there is more to seasonality than the simple effect of temperature variations. Furthermore, other critically important factors are involved, such as lockdown implementation, public education about hand hygiene and social distancing, quarantine for travellers and self-isolation. Some believe that with the arrival of spring and summer, the transmission of the virus will be reduced in the Northern Hemisphere, while it will rise in the Southern Hemisphere where low- and middle-income countries could face devastating strains on their healthcare systems [30].

We currently see in July 2020 an important increase of the virus propagation in several countries of the Southern Hemisphere in South America, Africa and Oceania. However, some countries in the Northern Hemisphere are still experiencing high COVID infection rates, such as the United States. Additionally, one has to take into account the intertropical convergence zone, in which epidemic seasonal patterns tend to *fade*.

The results presented here may suggest that SARS-CoV-2, despite its novelty, could possibly follow a similar pattern to that of other coronaviruses, and most other respiratory viruses in general. This, in alongside other measures taken by states such as lockdown implementations and expansion of testing capacities, might dampen the impact of the virus while possible therapies and vaccines are being developed.

## Data Availability

All data used in this paper are publicly available in the various countries' health organisations and ministries websites – see references below. It is therefore possible to replicate our findings.

## Supplementary material

For supplementary material accompanying this paper visit http://dx.doi.org/10.1017/S0950268820001831. click here to view supplementary material
