# Supplementary material for: COVID-19: relationship between atmospheric temperature and daily new cases growth rate
Source: Epidemiol Infect. 2020 Aug 19;148:e184. doi: 10.1017/S0950268820001831 (PMC7463156; doi:10.1017/S0950268820001831)
Supplement: Supplementary file 1 [file S0950268820001831sup001.docx]

**Appendix**

Legend: Global analysis of daily new cases growth rate (%) with regards to daily temperature (°C) in Lombardy (Italy) shows no correlation.
